# Bariatric Surgery as Treatment Strategy of Obesity in Saudi People: Effects of Gut Microbiota

**DOI:** 10.3390/nu15020361

**Published:** 2023-01-11

**Authors:** Seham J. Alqahtani, Hanan A. Alfawaz, Nadine M. S. Moubayed, Wail M. Hassan, Ahmad T. Almnaizel, Noura M. S. Alshiban, Jawahir M. Abuhaimed, Mohammed F. Alahmed, Mosffer M. AL-Dagal, Afaf El-Ansary

**Affiliations:** 1 Department of Food Science & Nutrition, College of Food Science & Agriculture, King Saud University, Riyadh 11495, Saudi Arabia; 2 Botany and Microbiology Department, Science College, Female Campus, King Saud University, Riyadh 11495, Saudi Arabia; 3 Department of Biomedical Sciences, School of Medicine, University of Missouri Kansas City, Kansas City, MO 64108, USA; 4 Experimental Surgery and Animal Lab, College of Medicine, King Saud University, P.O. Box 2925, Riyadh 11461, Saudi Arabia; 5 Health Sector, King Abdulaziz City for Science and Technology (KACST), Riyadh 12354, Saudi Arabia; 6 Anatomy Department, College of Medicine, King Saud University, Riyadh 11495, Saudi Arabia; 7 Central Research Laboratory, Female Campus, King Saud University, Riyadh 11495, Saudi Arabia

**Keywords:** obesity, gut microbiota, bariatric surgery, Laparoscopic Sleeve Gastrectomy (LSG)

## Abstract

Obesity prevalence is rising globally, as are the number of chronic disorders connected with obesity, such as diabetes, non-alcoholic fatty liver disease, dyslipidemia, and hypertension. Bariatric surgery is also becoming more common, and it remains the most effective and long-term treatment for obesity. This study will assess the influence of Laparoscopic Sleeve Gastrectomy (LSG) on gut microbiota in people with obesity before and after surgery. The findings shed new light on the changes in gut microbiota in Saudi people with obesity following LSG. In conclusion, LSG may improve the metabolic profile, resulting in decreased fat mass and increased lean mass, as well as improving the microbial composition balance in the gastrointestinal tract, but this is still not equivalent to normal weight microbiology. A range of factors, including patient characteristics, geographic dispersion, type of operation, technique, and nutritional and caloric restriction, could explain differences in abundance between studies. This information could point to a novel and, most likely, tailored strategy in obesity therapy, which could eventually be incorporated into health evaluations and monitoring in preventive health care or clinical medicine.

## 1. Introduction

Obesity has become the most prevalent health problem worldwide and has reached an epidemic proportion in society [1]. Obesity not only impairs health but also places an increased burden on the health care system as well. According to the WHO, 1.9 billion adults worldwide were overweight while 650 million were obese in 2016 [2]. The prevalence of obesity almost tripled: currently 39% of adults around the world are overweight and 13% are obese [2]. WHO estimates that by 2025, approximately 167 million people, adults and children, will become less healthy because they are overweight or obese [2]. The maximum proportions were observed in the Middle East and North Africa; for example, in Saudi Arabia, approximately 70 % of both gender were overweight and 35% were obese [3].

Obesity involves a complex interaction of genetic, lifestyle, and environmental factors. The gut microbiota is another potential factor that could contribute to the mechanisms of obesity and metabolic syndrome [4,5]. Research found a relationship between the human gut microbiota and obesity [6,7,8,9]. Obesity could possibly distress the gut microbiota composition and shift it to dysbiosis. The dysbiotic metabolic properties can enhance the body’s capacity to absorb energy from food and store it in the adipose tissue [9]. In addition, dysbiosis may increase intestinal permeability, which leads to a low-grade systemic inflammatory status [9,10,11].

Common microbiota in the gastrointestinal tract are bacteria, but viruses and fungi are still existing. The quantity of microbes in the gastric mucosa is estimated to be more than 100 trillion cells [12]. Although individuals have a unique microbiota composition, the gut microbiota is primarily members of four phyla (*Firmicutes*, *Bacteroidetes*, *Actinobacteria*, and *Proteobacteria*) [8]. Many factors affect the compositions and diversity of microbiota such as environmental and genetic factors, diet, probiotics, medications, and bariatric surgery [1,12,13,14]. According to the assessment of the literature, the most prevalent bacterial groups in the feces of people with obesity and normal weight belonged to the phyla *Firmicutes* and *Bacteroidetes*. Gram-positive *Firmicutes* bacteria (*Lactobacillus* spp. and *Clostridium leptum*, as well as *Clostridium coccoides*) and Gram-negative *Bacteroides* spp. made up a median proportion of 96.3% of all detected bacteria in various studies using either the universal primer or high-throughput sequencing methods [15,16]. The *Actinobacteria* phylum is less numerous and is mostly represented by the *Bifidobacterium* genus. The *Proteobacteria* phylum is mostly represented by *Escherichia coli*, which is found in a healthy gut and has been observed to rise following bariatric surgery. When compared to NGS-based metagenome profiling data, the qRT-PCR method is capable of quantifying these prevalent microorganisms [17].

Bariatric surgeries are the most effective approach to obesity management. They successfully sustain weight loss and improve associated co-morbidities over time [7,18,19,20]. Bariatric surgery is known as an effective method of weight reduction such that patients could conserve weight loss for more than 5 years [21]. The most commonly performed bariatric procedures are Roux-en Y Gastric Bypass (RYGB) and Laparoscopic Sleeve Gastrectomy (LSG) [20]. Globally, the LSG is becoming increasingly popular in clinical practice and provides comparable weight loss to the RYGB [22,23,24]. The LSG is gaining popularity in Saudi Arabia as well in the last five years according to recent survey executed by Al-Enazi and Al-Falah in 2017 [25].

Bariatric surgeries could affect the gut microbiota composition by inducing alterations in both environmental and systemic factors, as well as anatomical changes in the digestive tract [26]. Additionally, the metabolic improvement that happens post-surgery cannot be explained only by the caloric control and the weight loss alone but also by the changes in the microbial community. Although the changes of fecal microbiota composition may be caused by the microbial adaptation to caloric control, bariatric surgeries lead to a decrease in energy harvest by alterations in fermentation activity and the subsequent sustainable weight loss [8,27]. These alterations may express significant metabolic importance and could play a role in weight loss post-bariatric surgery.

The relative impact of these types of bariatric surgery on the composition and function of human gut microbiota is unclear. Animal data support the claim that changes in the gut microbiota contribute to the reduced host weight and adiposity after bariatric surgery and improved metabolic biomarkers [28,29]. However, there is a shortage in human studies that focus on bariatric surgery and its effect on intestinal microbiota; moreover, most of the work on intestinal microbiota were completed after RYGB compared to LSG [30,31,32]. Additionally, most of the studies have not investigated the alterations in gut microbiota composition and diversity before and after surgery [4,33].

To our knowledge, there are no studies being conducted in the Saudi population on the impact of the LSG and its effect on gut microbiota. So, this study will compare the impact of LSG on gut microbiota in patients with obesity pre- and post-surgery. These results will provide new insights regarding gut microbiota changes in Saudi patients after LSG. This information may indicate novel and most likely individualized interventions in obesity therapy, which could become incorporated into health assessments and monitoring in preventive health care or clinical medicine.

## 2. Materials and Methods

### 2.1. Study Participants

Candidates for bariatric surgery aged 19–60 years were recruited according to the international criteria for bariatric surgery, namely BMI ˃ 40 kg/m^2^ or ˃ 35 kg/m^2^ with some comorbidities. To act as a lean control group, ten healthy volunteers with normal weights (BMI 18.5–24.9) who lived in the same geographical area as the patients were selected. Recruitment occurred between January and July of 2021 at King Khalid University Hospitals (KKUH). All patients who came to the bariatric clinic during that time frame were screened for study eligibility and whoever matched our inclusion criteria and agreed to participate were included in this study. Only 34 participants met the requirements and agreed to take part in the study.

a. The exclusion criteria:

Individuals who were aged less than 18 years or were pregnant, with previous major gastrointestinal surgery, history of allergic/neurological/mental disease, history of colon cancer, inflammatory bowel disease, acute or chronic diarrhea in the last 2 months, and treatment with an antibiotic, pre- or probiotic agents in the 3 months before fecal sampling were excluded from the study.

b. Ethical approval of the study:

This work was approved by the Institutional Review Board (IRB) (Ref. No. 21/0084/IRB project No. E-20-5432) of the Medical College at King Saud University. All participants received informed consent forms, which were obtained concurrently with sample collection.

### 2.2. Study Design and Data Collection

An observational cohort prospective study was conducted for 12 months at KKUH in Riyadh. Clinical and biological samples were taken before LSG (basal or M0) and at 3, 6, and 12 months after surgery. At each time point, all participants were interviewed face-to-face to answer the questionnaire. The questionnaire included demographic data, including age, sex, and education level, and marital status, income per month, smoking status, and physical activity. It also included anthropometric data (weight, height, and waist and hip circumferences). Additionally, a bioelectrical impedance analysis used to assess body composition using electrical tissue conductivity and provided estimates of percent of water, body fat, and lean body mass (BIA Model 450 Bio impedance Analyzer; Biodynamics, Inc., Shoreline, WA, USA). The researcher assessed the participant’s usual food intake by using a 24 hours food-recall questionnaire over the previous 3 days of each visit.

There were 4 repeated visits for participants who underwent the sleeve process which had the same procedures (as mentioned below) for each visit. However, for participants of the control group, there was only one visit during their participation in this research study. The procedure for each visit was as follows:

1. Participants were interviewed face-to-face at the clinic to complete the questionnaire;
2. Body measurements were taken by the researcher (such as height, weight, waist and hip circumferences with height- and weight-measuring devices;
3. Body composition using electrical tissue conductivity was assessed to obtain an estimation of body fat present, lean body mass, and body water percent;
4. Blood samples were taken to check the level of lipid profile and glucose;
5. Stool samples were provided by participants to examine the microbiota composition.

According to their doctors, the postoperative diet progression involved a progressive return to solid foods (liquids, pureed, soft solids, and ultimately conventional foods) over the course of no more than 8 weeks. None of the subjects who received LSG were given any preoperative dietary recommendations.

### 2.3. Fecal Samples’ Collection and DNA Extraction

At each time point, stool samples were collected at the clinic under aseptic conditions with clean, sterile dry screw-top containers and treated immediately in the laboratory as 200-mg aliquots and stored at −20 °C until further analysis.

For DNA extraction, the QIAamp DNA Stool Mini Kit (Qiagen, Hilden, Germany) was used following the manufacturer’s instruction protocol. The DNA concentration and its molecular size was estimated by using a Nano-drop spectrophotometer (Thermo Scientific, San Diego, CA, USA).

### 2.4. Analysis of Fecal Microbiota by Real-Time PCR

Specific primers targeting different bacterial genera was used to characterize the fecal microbiota by real-time qPCR (see Table 1). RT-qPCR using SYBR Green was adapted to quantify the total bacteria population in addition to the dominant (>1% of fecal bacteria) bacterial species *Clostridium leptum* (*C. leptum*), *Clostridium coccoides* (*C. coccoides*), *Bacteroides*/*Prevotella*, and *Bifidobacterium*, the *Lactobacillus*/*Leuconostoc*/*Pediococcus* and for the subdominant bacterial species *Escherichia coli* (*E. coli*), as well as for the *Faecalibacterium prausnitzii* (*F. prausnitzii*).

RT-qPCR was performed using an ABI 7000 Sequence Detection System with software version 1.2.3 (Applied Biosystems, Foster City, CA, USA). Amplification and detection were carried out in 96-well plates with SYBR Green PCR 2× Master Mix (Applied Biosystems). Each reaction was run in duplicate in a final volume of 20 ml with 0.2 mmol/L final concentration of each primer and 10 μl of appropriately diluted DNA samples. Amplifications was carried out using the following ramping profile: one cycle at 95°C for 2 min, followed by 40 cycles of 95°C for 15 s, 60 °C for 1 min. following the manufacturer’s instruction protocols. Delta–delta Ct data were converted to 2 raised to the power of minus delta–delta Ct (2^−ΔΔCt^) to obtain the expression value.

### 2.5. Statistical Analysis

Continuous variables were summarized using mean ± one standard deviation. Categorical variables were described as number (*n*) and percentage (%). Temporal changes in gut microbiota were evaluated over the 1-year following surgery using repeated measures ANOVA and MANOVA when focusing on one dependent variable and more than one dependent variable, respectively. Both tests were performed using IBM SPSS v. 27 (IBM Corporation, Armonk, NY, USA). We also performed a paired *t*-test to evaluate the change pre- and 3, 6, and 12 months post-surgery. The *t*-test was performed in Microsoft Excel. The threshold of significance was defined as *p* < 0.05. Discriminant analysis (DA) and multinomial logistic regression (MLR) were performed using IBM SPPS v. 27.

## 3. Results and Discussion

### 3.1. Participants and Demographic Characteristics

Thirty-four patients, nineteen females and fifteen males, with morbid obesity who were candidates for LSG were enrolled in the study at M0. Five patients were excluded for the reason of not performing the surgery due to hyperglycemia (three participants), pregnancy (one participant), or contracting COVID-19 (one participant). In addition, one patient was lost to follow-up. Therefore, a total of 28 patients, 13 (46.4%) females and 15 (53.6%) males, completed the study until M12. In addition, we enrolled 11 healthy participants of a normal weight as the control group. The demographic characteristics of the surgery and control groups are presented in Table 2.

### 3.2. Anthropometric Characteristics and Clinical Data

LSG resulted in a significant change in body weight at M0 (121.31 ± 16.35 kg) and BMI (44.07 ± 5.25 kg/m^2^), to M3, weight and BMI were (98.70 ± 13.52 kg and 36.01 ± 5.47 kg/m^2^), respectively, and at M12, weight and BMI were 80.70 ± 11.65 kg and 29.40 ± 5.87 kg/m^2^, respectively (see Table 3). For the majority of parameters, improvement occurred rapidly in the first 3 months after surgery. At 12 M, the participants had lost 33 % of their initial weight.

The analyses of anthropometric data significantly changed during the one year immediately following surgery by using one-way repeated measures MANOVA (multivariate analysis of variance). The Wilks’ Lambda *p* value was <0.05 indicating a highly significant change in the combined clinical data as opposed to each alone. One-way repeated measures ANOVA was used to determine the significance of changes in individual analyses and the *p* values were significant in weight, BMI, Hip, Waist, Body fat, Muscle, and water percent, except for waist to hip ratio where the change was not significant (see Table 3).

The level of all biochemical indices measured in this study was improved with ALT, BUN, creatinine, HgbA1C, and fasting blood glucose showing a significant decrease over the 12-month follow-up period. The changing patterns were statistically significant (*p* < 0.05) for all mentioned variables, except for AST (Table 4). With weight loss, all circulating metabolic biomarkers improved significantly except for cholesterol and LDL (Table 4).

### 3.3. Changes of Gut Microbiota Composition

The average count for each bacterial group of the control and surgery groups are presented in Table 5. The *Bacteroides*/*Prevotella* group was significantly lower in the surgery group (*p =* 0.00) compared to the normal weight group. However, the population of *E. coli* was significantly higher in the surgery group compared to the normal weight (*p <* 0.05). In addition, the abundance of *C. leptum*, *C. coccoides*, and *F. prausnitzii* was lower in the surgery group with a significant level of (*p <* 0.001, *p <* 0.01, and *p <* 0.001), respectively.

However, at M3, all gut microbiota groups had a similar trend to that in the normal weight group, as no significant differences were found between them except for *E. coli* (*p =* 0.03) and *Lactobacillus* (*p* = 0.04). Meanwhile, at M6 post-surgery only the population of *C. leptum* and *F. prausnitzii* diminished significantly compared to the normal weight group (*p =* 0.00 and *p =* 0.01), respectively. On the other hand, after one year post-surgery, all the gut microbiota composition reduced significantly compared with the normal weight group except for the *E. coli* group that was still higher but with no significant differences. Details of *p*-value of all bacteria groups are presented in Table 5.

**Figure 1:**
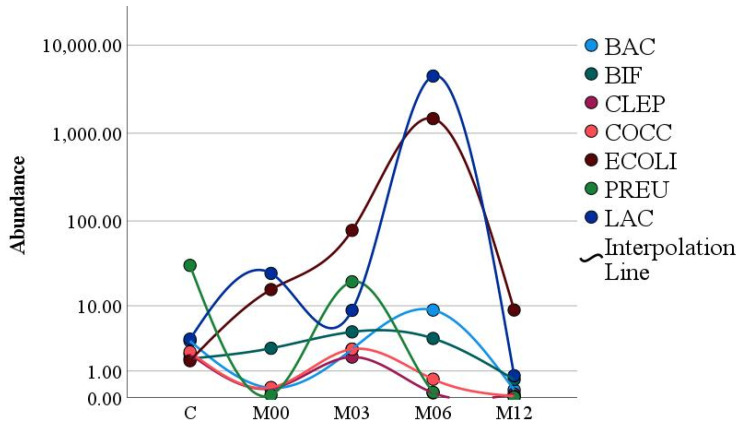
Microbiome dynamics during the first year following bariatric surgery (BAC = *Bacteroides*/*Prevotella*; BIF = *Bifidobacterium*; CLEP = *C. leptum*; COCC = *C. coccoides*; ECOLI = *E. coli*; PREU = *F. prausnitzii*; LAC = *Lactobacillus*/*Leuconostoc*/*Pediococcus*). (M00: pre-surgery; M03: 3 months post-surgery; M06: 6 months post-surgery; M12: 12 months post-surgery).

In the year following bariatric surgery, we demonstrate that the gut flora underwent a temporal shift (*p <* 0.005) (Figure 1; Table 6). The individual microbiome groups also altered over the course of the first year following surgery at *p* < 0.05 with the exception of Bacteroides/Prevotella which showed no significant change (*p* = 0.094) (Table 6).

For the pairwise differences between time points, the concentration of genera *C. leptum*, *E. coli*, *and F. prausnitzii* showed a significant increase at M3 post-surgery compared to pre-surgery (*p =* 0.04, *p =* 0.01, and *p <* 0.05), respectively. At M6 post-surgery, only *C. leptum* diminished significantly compared to pre-surgery (*p =* 0.02). However, all abundance of gut microbiota diminished significantly at M12 post-surgery except for genera *Bacteroides*/*Prevotella* and *E. coli* did not show significant decreases. All detailed results presented in Table 6.

**Figure 2:**
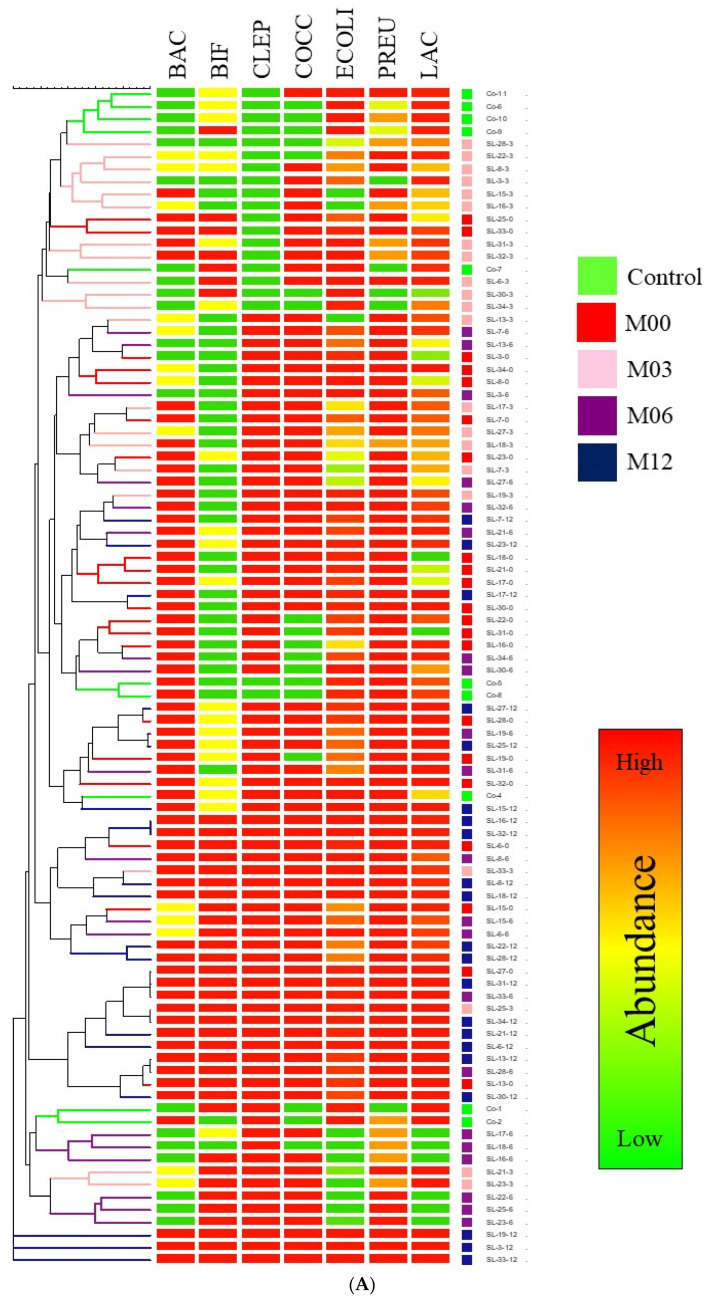

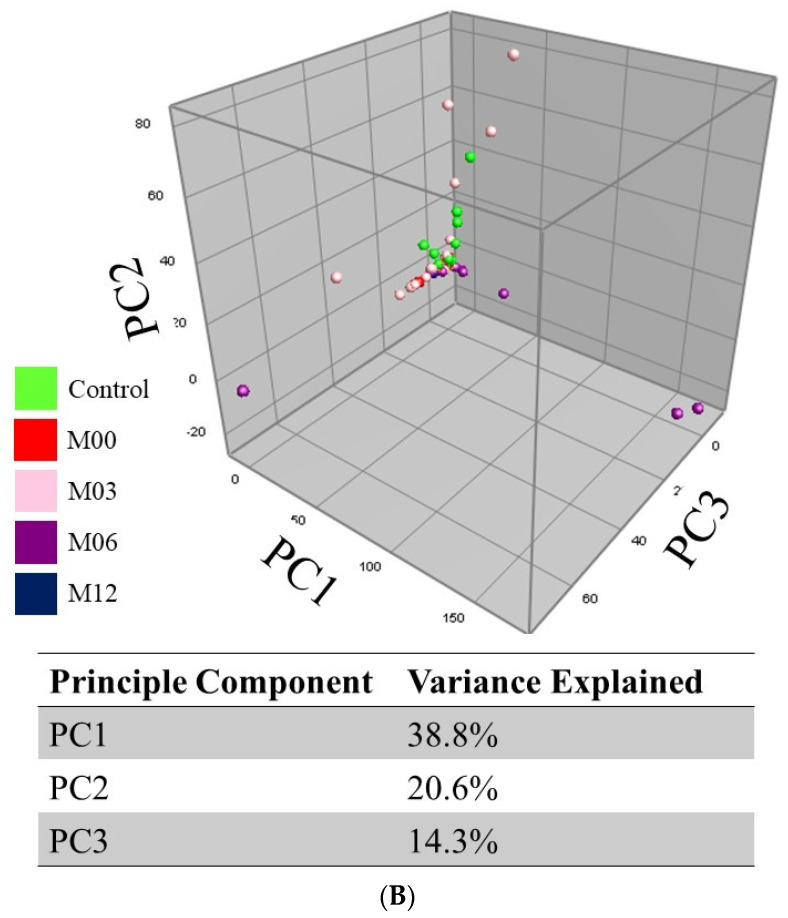
Unsupervised partitioning of gut microbiomes of healthy controls and surgery patients before surgery, 3 months, 6 months, and 1 year after surgery. Data were analyzed using hierarchical clustering (**A**) and principal component analysis (**B**). M00: pre-surgery; M03: 3 months post-surgery; M06: 6 months post-surgery; M12: 12 months post-surgery.

We then wanted to investigate the relationships between microbiomes of surgery patients at various time points after surgery and how they compared to the control participants. Hierarchical clustering and PCA (Figure 2) failed to separate the gut microbiome groups, implying that the microbiomes at these time points and from the control participants were not distinct enough to facilitate such a separation. It is noteworthy that the microbiome of the surgery patients before surgery did not cluster with control participants, indicating distinct microbiomes between the two groups.

**Figure 3:**
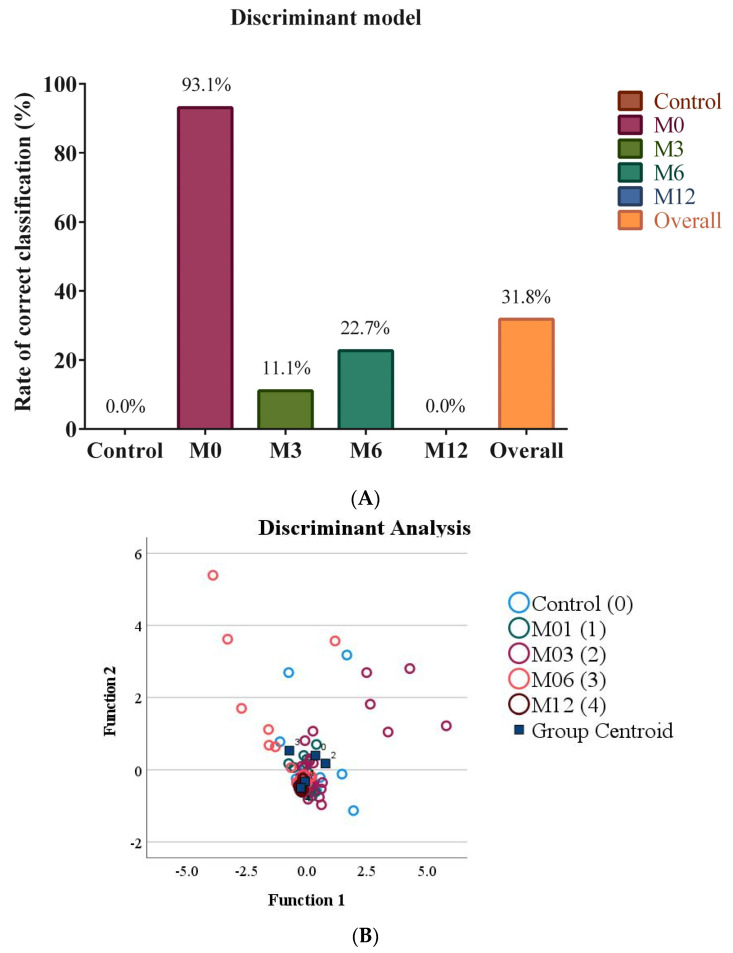
Discriminant analysis representing the gut microbiomes of healthy control participants and bariatric surgery patients before surgery and 3 months, 6 months, and 12 months after surgery. The rates of correct classification (**A**) and a scatter plot (**B**) are shown. (M0: pre-surgery; M3: 3 months post-surgery; M6: 6 months post-surgery; M12: 12 months post-surgery).

Since partitioning in hierarchical clustering and PCA does not take into account group membership, we were interested in analyzing the data using methods designed to maximize group separation, such as discriminant analysis and binary logistic regression. A DA model with 38.1% of variance explaining group membership (Wilks’ Lambda of 0.619) was statistically significant (*p* = 0.008). However, the discriminant model was not able to distinguish between microbiome groups, as demonstrated by low eigenvalues and canonical correlations, even for the first and second discriminants (Table 7), and low RCCs (Figure 3A). As expected, a scatter plot based on the first and second discriminant functions showed a substantial overlap between groups (Figure 3B). The contribution of each microbiome group to each of the discriminant functions (standardized canonical discriminant function coefficients) are shown in Table 8.

Table 8 represents how much each of the microbiome groups contributed to each of the discriminant functions. In function 1, *E. coli* has 4.140 and *Bacteroides* has −3.304. So, these two are the most influential groups for this variable. *C. coccoides* and *Lactobacillus* has relatively large numbers as well, which means they also contribute, but less than *E. coli* and *Bacteroides.* In the corresponding scatter plot (Figure 3B), function 1 is on the x axis, meaning any group separation on the x axis is mostly based *E. coli*, *Bacteroides*, *Lactobacillus*, and *C. coccoides*, in descending order of importance.

**Figure 4:**
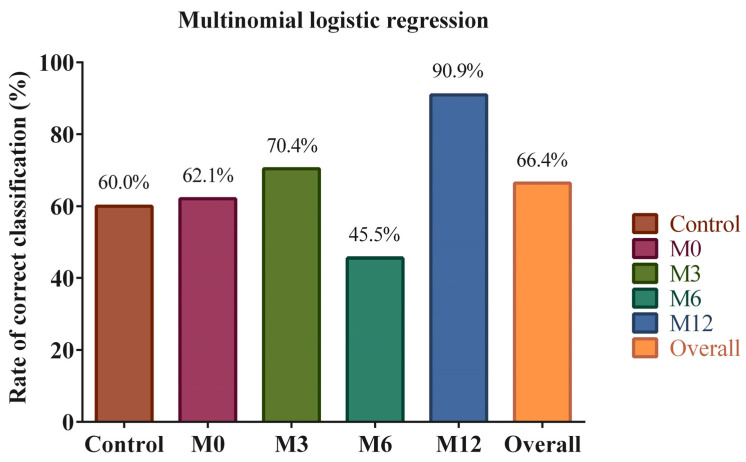
Rates of correct classification from a multinomial logistic regression model representing the gut microbiomes of healthy control participants and bariatric surgery patients before surgery and 3 months, 6 months, and 12 months after surgery. M0: pre-surgery; M3: 3 months post-surgery; M6: 6 months post-surgery; M12: 12 months post-surgery.

We then attempted MLR, which is often more powerful than DA. The model represented a statistically significant improvement over the null hypothesis (*p* < 0.001), fitted the data very well with both Pearson chi-square and Deviance chi-square *p* values equal to 1, had a Nagelkerke pseudo-R2 of 0.823 (Table 9), and an overall RCC (RCC calculated over all participants and time points) of 66.4% (Figure 4). RCCs of individual surgery patients’ time points and healthy controls ranged from 45.5% to 90.9% (Figure 4).

We then wanted to determine the significance and contribution of each of the variables to the model. Variables that contributed significantly to the overall model (i.e., all variables at all time points) were *C. leptum*, *C. coccoides*, *E. coli*, *F. prausnitzii*, and *Lactobacillus* (Table 10). Individual variable contributions were evaluated based on their coefficients (natural log of odds ratios) and odds ratios. The odds ratio represents the ratio of the odds of falling in one of the time points of surgery patients (M0 through M12) to the odds of falling in the control group. It is important to remember that strong predictor variables are the ones with large—in absolute value, whether positive or negative—coefficients and odds ratios substantially above or below 1. In addition, a *p* value was assigned to each variable to indicate whether it was a reliable predictor of surgery group time points as opposed to the control. Our results show that only *F. prausnitzii* for M6 and M12 and *C. leptum* for M6 were significant (Table 11). A negative sign of the coefficient and a very small odds ratio indicate that a low abundance of *F. prausnitzii* is the strongest predictor of M0, M6, and M12. The top predictors, in descending order of importance, were *F. prausnitzii* and *C. leptum* for M0; *F. prausnitzii*, *C. leptum*, *C. coccoides*, and *Bacteroides* for M6; and *F. prausnitzii*, *C. coccoides*, *C. leptum*, and *Bacteroides* for M12. M3 did not have strong predictors (Table 11). Overall, we concluded that gut microbiomes of bariatric surgery patients before and after surgery and those of healthy controls were different enough to achieve a modest-to-poor separation.

Despite the small number of participants, this study compared the makeup of the gut microbiota between obese and lean persons. In addition, it presented alterations in the Saudi population for the first time in the same individuals before and after LSG over a one-year follow-up. According to recent research, blood biomarkers and gut bacteria are crucial for maintaining the health of the host [34,35]. Important information about a possible connection between gut microbiota composition and metabolic adaptation came from the analysis of the kinetic change following LSG [35].

It was found that the abundance of *E. coli* intestinal flora was significantly increased post-surgery showing a maximum abundance after 12 M. (Figure 1). Based on the fact that biomarkers act as quantifiable indicators of a biological condition or exposure to the environment [36]. Genera such as *Bacteroides*/*Prevotella* and *Bifidobacterium* may be useful in this regard as indicators of food- and lifestyle change post-surgery [37,38]

Our data (Table 3 and Table 4) revealed that the significant weight loss and metabolic improvements a year after LSG are comparable to those noted by other sources [35,39,40]. This remarkable improvement in glucose, HbA1c, lipid profile, ALT, AST, BUN, and creatinine could be attributed to the rapid loss of weight, decrease in caloric intake, and management of lipid profiles in the body following surgery.

Although the participants overall had successfully lost 33 % of their initial weight at M12, male participants lost more weight than females as their mean BMIs were 25.80 (2.79) and 32.52 (6.11)), respectively (Table 3). This result was attributed to our observations that some female patients were suffering unconsciously from lipedema so the surgery could help a little but is not the whole solution as surgery will lead to weight loss in the upper body without changing the areas primarily affected by lipedema which is the lower body. Therefore, whilst there is a growing consciousness of this condition, its diagnosis can still be challenging among physicians [41].

Most individuals after bariatric surgery had rapid alterations in the variety and makeup of their gut microbiomes. Previous research using human and animal models has shown that bariatric surgery alters the composition of the gut flora [28,29,34,40,41].

In the present study, the abundance of the gut microbiome composition as a whole significantly changed during the year immediately following surgery. There was a trend toward an increase in gut microbiota composition at M3, with a significant decrease at M12 post-surgery. In our samples, there was a greater abundance of *Bacteroides*, *Bifidobacterium*, *C. leptum*, *C. coccoides*, *E. coli*, and *F. prausnitzii* observed at M3 compared to pre-surgery; subsequently, all of them diminished significantly at M12 except for the *Bacteroides* groups that decreased without significance. It has been explained in previous studies that the *Firmicute* groups are more capable of harvesting energy from nutrients hence stimulating more absorption of calories which increases weight compared to the *Bacteroidetes* phylum [5]. Therefore, bariatric surgery could lead to the restoration of a proper microbial balance in the gastrointestinal tract. Studies performed on animals have found a lower abundance of *Firmicutes* and higher *Bacteroidetes* among the SG group compared with the RYGB group. However, this was in contrast with our data as we found a decrease in the abundance of both *Bacteroidetes* and *Firmicutes*. In a human study that followed the participants for 6 months following SG, the data demonstrated that the abundance of *Bacteroidetes* dropped, which contrasts with our results at the same duration post-surgery, but after 12 months our data showed a similar trend [35]. However, our data for *Firmicutes* proportions after 6 and 12 months of surgery were comparable to theirs.

The majority of studies to date have concentrated on the effect of the Roux-en-Y gastric bypass (RYGB) on gut microbiota. Given that the LSG anatomic rearrangements differ from RYGB, the effects on gut microbiota composition differ. For example, in a recent study that compared both surgeries, they found that the phylum *Bacteroidetes* was under-represented in LSG subjects compared to RYGB; the reasons for this were due to physiological changes associated with these treatments and their consequences, which may explain these differences [35]. Moreover, these bacteria are also favored by less acidic luminal pH [42]. Our data over one year of LSG are in accordance with these findings. In addition, an important increase in *E. coli* was observed in this study which are in agreement with previous studies that have shown clear alterations in the *Proteobacteria* such as *E. coli* in both type of surgeries [30,35,39,43]. The explanation of this alteration could be associated with an increase in luminal acidity and dissolved oxygen after these procedures, as well as *E. coli*’s ability to harvest energy efficiently after BS during the initial nutritional starvation [42]. Additionally, it has to be noted that some patients are using proton pump inhibitors (PPIs) after surgery to suppress gastric acid production and some of them continue to use them for the first 3 to 4 months. A review study found an association between PPI intake and increased colonization of the distal intestine by gastric bacteria, such as *E. coli* [44]. This factor could affect our results when the PPIs were discontinued, hence may have had an influence on the abundance of some microbiome composition such as *E. coli* species.

Furthermore, in our sample, we found significant changes in all individuals’ gut microbiota as mentioned above in the obesity group at M0 compared to the normal weight group except for the *Bifidobacterium* and *Lactobacillus* group. These results are in accordance with the first study completed by Ley et al. in humans in 2006 as they found that people with obesity had a reduction in the abundance of *Bacteroidetes* compared to people of a normal weight [45]. However, our results are contradictory with a study performed by Yasir et al., in the Saudi population in 2015, as they found no differences in the richness and biodiversity between the obese and normal weight groups and no differences in *Bacteroidetes* between both groups. However, they revealed that Saudis with obesity possessed more *Firmicutes* than those of a normal weight [46]. After one year post-surgery, gut microbiota composition reduced significantly comparing with those of a normal weight except for the *E. coli* group. Therefore, we concluded that the gut microbiota of patients who underwent surgery and lost 33% of their initial weight failed to restore to the levels of similar microbiota of normal weight people.

## 4. Conclusions

In conclusion, the LSG may enhance the metabolic profile, which contributes to decreased fat mass and increased lean mass, as well as improving the microbial composition; however, this is still not comparable to the microbiological composition of people of normal weight. Variations in abundance between studies could be attributed to a variety of factors, including subject characteristics, geographic dispersion, type of surgery, methodology, and dietary and caloric restriction. Because this is an observational study, more functional investigations are needed to understand the role of the gut microbiota in weight reduction and the metabolic benefits reported after LSG, which may eventually aid in the development of microbiome-based medicines aimed at treating metabolic disorders. In this study, fecal microbiota analysis was limited to seven 16S rRNA-gene-targeted specific primers and cannot provide comprehensive results on the role of LSG on the gut microbiota alterations, which is considered a limitation in our study. However, this is the first study in Saudi Arabia to indicate changes in gut microbiota composition in obese adults before surgery, and 3, 6, and 12 months after surgery.

## Figures and Tables

**Table 1:** Primers that were used for the Real-Time PCR: (*).

Total Organism	Primers	Sequence 5′–3′
All bacteria	*F_Bact 1369*	CGG TGA ATA CGT TCC CGG
	*R_Prok 1492*	TAC GGC TAC CTT GTT ACG ACT T
*C. leptum*	*F_Clept 09*	CCT TCC GTG CCG SAG TTA
	*R_Clept 08*	GAA TTA AAC CAC ATA CTC CAC TGC TT
*Bifidobacterium*	*F_Bifid 09c*	CGG GTG AGT AAT GCG TGA CC
	*R_Bifid 06*	TGA TAG GAC GCG ACC CCA
*C. coccoides*	*F_Ccoc 07*	GAC GCC GCG TGA AGG A
	*R_Ccoc 14*	AGC CCC AGC CTT TCA CAT C
*Bacteroides*/*Prevotella*	*F_Bacter 11*	CCT WCG ATG GAT AGG GGT T
	*R_Bacter 08*	CAC GCT ACT TGG CTG GTT CAG
*E. coli*	*E. coli F*	CAT GCC GCG TGT ATG AAG AA
	*E. coli R*	CGG GTA ACG TCA ATG AGC AAA
*Lactobacillus*/*Leuconostoc*/*Pediococcus*	*F_Lacto 05*	AGC AGT AGG GAA TCT TCC A
	*R_Lacto 04*	CGC CAC TGG TGT TCY TCC ATA TA
*F. prausnitzii*	*F_prau 07*	CCA TGA ATT GCC TTC AAA ACT GTT
	*F_prau 02*	GAG CCT CAG CGT CAG TTG GT

* Reference [30].

**Table 2:** The descriptive characteristics of the surgery and control groups.

	Control (*n*) %	Pre-Surgery (*n*) %
Participants (No.)	11	28
Gender		
Male	(2)18.2	(13) 46.4
Female	(9) 81.8	(15) 53.6
Age group		
19–30 y	(10) 90.9	(15) 53.6
31–40 y	(1) 9.1	(5) 17.9
41–50 y	0	(5) 17.9
˃50	0	(3) 10.7
Marital status		
Single	(9) 81.8	(12) 42.9
Married	(2) 18.2	(15) 53.6
Divorced	0	(1) 3.6
Edu. Level		
Primary or less	0	(2) 7.1
Secondary	0	(9) 32.1
Graduate	(10) 90.9	(15) 53.6
Postgraduate.	(1) 9.1	(2) 7.1
Income level (SAR)		
˂5000	(5) 45.5	(9) 32.1
5000–10,000	0	(9) 32.1
˃10,000 to 15,000	0	(5) 17.9
˃15,000 to 20,000	(2) 18.2	(2) 7.1
˃20,000	(4) 36.4	(3) 10.7
Health Status		
Healthy	11	(19) 67.9
Sleep Apnea	0	(3) 10.7
HTN	0	(2) 7.1
CVD	0	(1) 3.6
Diabetes, HTN.	0	(1) 3.6
Diabetes, HTN, High cholesterol	0	(1) 3.6

**Table 3:** Anthropometric characteristics for controls and surgery group. Body mass index (BMI); waist-to-hip ratio (WHR).

	Control	M0	M3	M6	M12	*p*-Value *
	Mean (SD)	Mean (SD)	Mean (SD)	Mean (SD)	Mean (SD)	<0.05
Weight (kg)	54.64 (8.21)	121.31 (16.35)	98.70 (13.52)	87.23 (12.73)	80.70 (11.65)	<0.001
Male		129.4 (17.24)	101.5 (14.8)	86.73 (11.59)	78.93 (8.14)	
Female		114.26 (12.10)	96.2 (12.1)	87.66 (14.02)	82.23 (14.13)	
BMI (kg/m^2^)	21.59 (2.04)	44.07 (5.25)	36.01 (5.47)	31.79 (5.96)	29.40 (5.87)	<0.001
Male		42.28 (4.55)	33.3 (3.8)	28.54 (3.41)	25.80 (2.79)	
Female		45.60 (5.46)	38.3 (5.7)	34.60 (6.35)	32.52 (6.11)	
Waist (cm)	73.73 (8.43)	125.82 (12.56)	110.02 (11.25)	101.25 (8.53)	97.13 (8.51)	<0.001
Male		132.30 (12.63)	113.61 (12.49)	102.69 (10.27)	97.23 (8.24)	
Female		120.20 (9.71)	106.90 (9.37)	100.0 (6.80)	97.03 (9.01)	
Hip (cm)	96.36 (7.90)	141.50 (19.20)	123.14 (10.20)	115.07 (10.50)	110.25 (10.80)	<0.001
Male		139.15 (21.75)	118.69 (9.12)	109.73 (7.98)	103.76 (6.19)	
Female		143.53 (17.19)	127.00 (9.74)	119.70 (10.42)	115.86 (10.91)	
WHR (Ratio)	0.76 (0.07)	0.89 (0.10)	0.89 (0.10)	0.88 (0.08)	0.89 (0.09)	<0.631
Male		0.95 (0.08)	0.95(0.06)	0.93 (0.05)	0.93 (0.05)	
Female		0.84 (0.08)	0.84 (0.08)	0.84 (0.08)	0.84 (0.08)	
Body fat (%)	21.88 (5.96)	45.32 (5.83)	39.96 (6.69)	35.54 (8.23)	32.40 (9.56)	<0.001
Male		40.26 (2.67)	33.90 (3.25)	28.39 (4.00)	23.57 (3.73)	
Female		49.70 (3.87)	45.20 (3.71)	41.74 (5.30)	40.04 (5.42)	
Muscle mass	39.54 (7.74)	63.49 (11.22)	56.51 (8.91)	53.28 (7.28)	51.78 (7.18)	<0.001
Male		73.57 (6.75)	63.96 (6.35)	59.06 (5.40)	57.65 (4.46)	
Female		54.74 (5.22)	50.06 (4.73)	48.25 (4.34)	46.68 (4.74)	
Water (%)	55.67 (5.07)	39.23 (3.43)	42.76 (4.55)	45.80 (5.68)	48.23 (6.92)	<0.001
Male		42.06 (2.30)	46.63 (2.78)	50.54 (3.25)	54.48 (3.26)	
Female		36.76 (2.03)	39.40 (2.68)	41.68 (3.75)	42.81 (3.91)	

* All variables considered significant if *p*-value < 0.001.

**Table 4:** Clinical and biological characteristics. (ALT) Alanine Transaminase; (AST) Aspartate Transaminase; (BUN) Blood Urea Nitrogen; (FBG) Fasting Blood Glucose; (HDL) High-Density Lipoprotein; (LDL) Low-Density Lipoprotein.

	Control	M0	M3	M6	M12	*p*-Value
	Mean (SD)	Mean (SD)	Mean (SD)	Mean (SD)	Mean (SD)	<0.05
ALT	20.36 (7.42)	34.96 (22.39)	35.54 (19.48)	25.71 (12.60)	24.07 (6.49)	0.038
AST	14.55 (3.72)	19.07 (13.96)	23.96 (15.64)	15.75 (10.79)	15.64 (6.70)	0.133
BUN	3.54 (0.76)	4.39 (1.27)	3.01 (1.33)	3.72 (0.89)	4.14 (1.34)	<0.001
Creatinine	58.91 (10.47)	66.04 (14.64)	57.21 (9.81)	61.07 (9.78)	62.89 (11.56)	0.014
Hgb A1c	5.32 (0.17)	5.88 (0.50)	5.39 (0.40)	5.34 (0.36)	5.25 (0.33)	<0.001
FBG (mmol/L)	4.94 (0.39)	5.25 (0.88)	4.62 (0.64)	4.70 (0.67)	4.58 (0.57)	0.012
Total cholesterol (mmol/L)	4.02 (0.64)	4.61 (0.68)	4.49 (0.83)	4.74 (0.92)	4.82 (0.93)	0.103
HDL-cholesterol (mmol/L)	1.44 (0.42)	1.20 (0.25)	1.11 (0.21)	1.31 (0.26)	1.49 (0.28)	<0.001
LDL-cholesterol (mmol/L)	2.25 (0.38)	2.86 (0.68)	2.86 (0.79)	3.00 (0.90)	2.93 (0.75)	0.596
Triglyceride (mmol/L)	0.84 (0.17)	1.28 (0.72)	1.15 (0.33)	0.98 (0.20)	0.93 (0.28)	0.009

**Table 5:** Comparison of Bacterial composition between controls and surgery group over 12 months, M0: pre-surgery, M3: 3 months post-surgery, M6: 6 months post-surgery, M12: 12 months post-surgery.

	Control	M0	M3	M6	M12	*p* Value	*p* Value	*p* Value	*p* Value	*p* Value	*p* Value	*p* Value
	Mean (SD)	Mean (SD)	Mean (SD)	Mean (SD)	Mean (SD)	Co, M0	Co, M3,	Co, M6	Co, M12	M0, M3	M0, M6	M0, M12
All Bacteria	15.55 (0.63)	15.09 (0.97)	14.51 (1.57)	12.56 (2.54)	10.05 (1.47)	0.18	0.05	0.00	0.00	0.19	0.00	0.00
*Bacteroids*	3.38 (3.73)	0.34 (0.42)	3.03 (7.19)	8.85 (22.14)	0.22 (0.11)	0.00 *	0.89	0.45	0.00 *	0.09	0.09	0.23
*Bifidobacterium*	1.77 (2.11)	2.22 (3.30)	4.94 (6.98)	3.69 (9.57)	0.60 (1.01)	0.64	0.17	0.54	0.04 *	0.12	0.51	0.01 *
*C. leptum*	2.11 (2.53)	0.26 (0.19)	2.24 (4.27)	0.13 (0.11)	0.10 (0.05)	0.00 *	0.93	0.00 *	0.00 *	0.04 *	0.02 *	0.00 *
*C. coccoides*	2.30 (3.18)	0.30 (0.35)	3.11 (8.88)	0.62 (2.01)	0.03 (0.02)	0.01 *	0.78	0.08	0.00 *	0.16	0.50	0.00 *
*E. coli*	1.62 (1.74)	15.56 (0.16)	81.35 (113.05)	381.58 (913.83)	8.86 (12.38)	0.05 *	0.03 *	0.20	0.08	0.01 *	0.07	0.25
*F. prausnitzii*	4.28 (9.44)	0.05 (0.08)	2.57 (5.70)	0.15 (0.29)	0.01 (0.01)	0.00 *	0.53	0.01 *	0.04 *	0.05 *	0.17	0.03 *
*Lactobacillus*	1.88 (1.80)	14.59 (20.84)	8.81 (9.79)	875.06 (1657.47)	0.77 (1.13)	0.07	0.04 *	0.11	0.04 *	0.28	0.02 *	0.01 *

* *p*-value is significant at 0.05, from independent *t*-test between control and surgery and paired *t*-test between surgery groups.

**Table 6:** Temporal changes in gut microbiome after bariatric surgery.

Statistical Test	Microbiome Group	*p*
One-way repeated measures MANOVA	Whole microbiome	0.005
One-way repeated measures ANOVA	*Bacteroides*/*Prevotella*	0.094
	*Bifidobacterium*	0.002
	*C. leptum*	<0.001
	*C. coccoides*	0.004
	*E. coli*	0.034
	*F. prausnitzii*	0.023
	*Lactobacillus*/*Leuconostoc*/*Pediococcus*	<0.001

**Table 7:** Characteristics of a discriminant model representing gut microbiomes of healthy control participants and bariatric surgery patients before surgery and 3 months, 6 months, and 12 months after surgery.

Function	Eigenvalue	Canonical Correlation
1	0.293	0.476
2	0.163	0.375
3	0.070	0.256
4	0.004	0.060

**Table 8:** Standardized canonical discriminant function coefficients.

	Function			
	1	2	3	4
*E. coli*	4.140	−3.628	5.729	4.345
*Bacteroides*	−3.304	3.253	−4.011	−2.710
*Lactobacillus*	−1.978	1.590	−2.222	−1.659
*C. coccoides*	1.256	−0.781	1.254	1.047
*C. leptum*	0.692	0.204	−0.125	0.151
*F. prausnitzii*	0.449	0.014	−0.025	0.250
*Bifidobacterium*	0.140	0.538	0.528	−0.656

**Table 9:** Characteristics of a multinomial logistic regression model representing the gut microbiomes of healthy control participants and bariatric surgery patients before surgery and 3 months, 6 months, and 12 months after surgery.

Model Significance	Goodness-of-Fit Pearson	Goodness-of-Fit Deviance	Nagelkerke Pseudo-R2
*p* < 0.001	1.0	1.0	0.823

**Table 10:** Significance of variables in the MLR model.

Variable	Chi-Square	df	*p* Value
*Bacteroides*	5.642	4	0.228
*Bifidobacterium*	3.159	4	0.532
*C. leptum*	11.936	4	0.018
*C. coccoides*	11.148	4	0.025
*E. coli*	19.679	4	<0.001
*F. prausnitzii*	19.605	4	<0.001
*Lactobacillus*	10.426	4	0.034

**Table 11:** Multinomial regression model parameter estimates, M0: pre-surgery; M3: 3 months post-surgery; M6: 6 months post-surgery; M12: 12 months post-surgery.

Category	Variable	Coefficient	*p* Value	Odds Ratio	Lower Bound 95% CI	Upper Bound 95% CI
M0	*F. prausnitzii*	−4.047	0.148	0.017	7.24 × 10^−5^	4.221
	*C. leptum*	−1.35	0.202	0.259	0.033	2.062
	*Bacteroides*	−0.619	0.401	0.539	0.127	2.285
	*C. coccoides*	0.582	0.462	1.79	0.38	8.436
	*E. coli*	0.181	0.222	1.198	0.896	1.601
	*Lactobacillus*	0.035	0.488	1.036	0.938	1.144
	*Bifidobacterium*	−0.012	0.931	0.988	0.748	1.304
M3	*E. coli*	0.213	0.15	1.237	0.926	1.652
	*C. leptum*	0.15	0.368	1.162	0.838	1.611
	*C. coccoides*	0.079	0.631	1.082	0.784	1.493
	*Bacteroides*	−0.07	0.738	0.932	0.619	1.404
	*Bifidobacterium*	0.07	0.612	1.072	0.819	1.403
	*Lactobacillus*	0.007	0.901	1.007	0.906	1.119
	*F. prausnitzii*	−0.005	0.56	0.995	0.98	1.011
M6	*F. prausnitzii*	−13.657	0.033	1.17 × 10^−6^	4.25 × 10^−12^	0.324
	*C. leptum*	−7.181	0.031	0.001	1.13 × 10^−6^	0.514
	*C. coccoides*	1.596	0.301	4.931	0.24	101.13
	*Bacteroides*	0.847	0.365	2.333	0.372	14.615
	*E. coli*	0.183	0.217	1.201	0.898	1.605
	*Bifidobacterium*	0.101	0.479	1.106	0.837	1.462
	*Lactobacillus*	0.034	0.507	1.034	0.936	1.143
M12	*F. prausnitzii*	−53.65	0.013	5.01 × 10^−24^	1.79 × 10^−42^	1.40 × 10^−5^
	*C. coccoides*	−22.476	0.059	1.73 × 10^−10^	1.28 × 10^−20^	2.344
	*C. leptum*	−5.054	0.295	0.006	4.99 × 10^−7^	81.635
	*Bacteroides*	1.847	0.335	6.343	0.148	271.813
	*Lactobacillus*	−0.25	0.382	0.779	0.445	1.364
	*Bifidobacterium*	−0.248	0.414	0.78	0.43	1.416
	*E. coli*	0.205	0.177	1.227	0.912	1.651

## Data Availability

The datasets of the current study are available from the corresponding author on reasonable request.
